# Association between different obesity phenotypes and hypothyroidism: a study based on a longitudinal health management cohort

**DOI:** 10.1007/s12020-021-02677-2

**Published:** 2021-04-05

**Authors:** Yupeng Wang, Haiyan Lin, Qihang Li, Liying Guan, Meng Zhao, Fang Zhong, Jing Liu, Zhongshang Yuan, Honglin Guo, Yongfeng Song, Ling Gao, Jiajun Zhao

**Affiliations:** 1 Department of Endocrinology, Shandong Provincial Hospital, Cheeloo College of Medicine, Shandong University, Jinan, Shandong China; 2 Shandong Provincial Key Laboratory of Endocrinology and Lipid Metabolism, Institute of Endocrinology and Metabolism, Shandong Academy of Clinical Medicine, Jinan, Shandong China; 3 Shandong Clinical Medical Center of Endocrinology and Metabolism, Jinan, Shandong China; 4 Health Management Center, Shandong Provincial Hospital Affiliated to Shandong First Medical University, Jinan, Shandong China; 5 Department of Endocrinology, Shandong Provincial Hospital Affiliated to Shandong First Medical University, Jinan, Shandong China; 6 Department of Biostatistics, School of Public Health, Shandong University, Jinan, Shandong China; 7 Department of Scientific Center, Shandong Provincial Hospital Affiliated to Shandong First Medical University, Jinan, Shandong China

**Keywords:** Obesity phenotype, Hypothyroidism, Sex difference, Age difference, Generalized estimating equation method

## Abstract

**Purpose:**

Obese individuals have an increased risk of hypothyroidism. This study investigated the sex-specific association between obesity phenotypes and the development of hypothyroidism.

**Methods:**

The study population was derived from a health management cohort in Shandong Provincial Hospital from 2012 to 2016. In total, 9011 baseline euthyroid adults were included and classified into four groups according to obesity phenotype: metabolically healthy nonobese (MHNO), metabolically healthy obese (MHO), metabolically unhealthy nonobese (MUNO), and metabolically unhealthy obese (MUO). The median follow-up time was 1.92 (1.00–2.17) years. Incidence density was evaluated and a generalized estimation equation method was used to investigate the associations between obesity phenotypes and the development of hypothyroidism.

**Results:**

The incidence densities of hypothyroidism in males with a consistent obesity phenotype were 12.19 (8.62–16.76), 15.87 (11.39–21.56), 14.52 (6.74–27.57), and 19.88 (14.06–27.34) per 1000 person-years in the MHNO, MHO, MUNO, and MUO groups, respectively. After adjusting for confounding factors, compared with the MHNO phenotype, the MHO, MUNO, and MUO phenotypes were independent risk factors for developing hypothyroidism in males. In the subgroup analysis, the MHO and MUO phenotypes were independent risk factors for developing hypothyroidism in males under 55 years, while the MUNO phenotype was an independent risk factor in males over 55 years. The MHO, MUNO, and MUO phenotypes were not independent risk factors for hypothyroidism in females.

**Conclusion:**

Both obesity and metabolic abnormities are associated with a higher risk of hypothyroidism in males. The underlying mechanism of the sex and age differences in this association needs further investigation.

## Introduction

Hypothyroidism refers to the common pathological condition of thyroid hormone deficiency and includes overt hypothyroidism and subclinical hypothyroidism [1]. In China, the prevalence of overt hypothyroidism is 1.02%, and the prevalence of subclinical hypothyroidism increased from 3.21% in 1999 to 12.93% in 2017 [2]. Many studies have found that subclinical hypothyroidism is associated with an increased risk of cardiovascular disease events and mortality [3, 4]. These facts highlight the need to identify the risk factors for hypothyroidism to prevent the increase in its incidence.

Obesity is also a global health problem, with growing incidence worldwide and serious adverse health effects. In recent years, the relationship between obesity and thyroid dysfunction has drawn increased attention, and it has been suggested that obesity might not be only the result but also the cause of thyroid dysfunction [5–7]. A meta-analysis including 22 studies showed that the obese population had increased risks of both overt hypothyroidism and subclinical hypothyroidism [8]. Obesity is typically associated with a constellation of metabolic abnormities, such as dyslipidemia, hypertension, and hyperglycemia. A prospective cohort study found that participants with metabolic syndrome at baseline had an increased risk of developing subclinical hypothyroidism [9].

Although the deleterious metabolic effects of obesity are well recognized, metabolic responses to obesity differ at the individual level [10]. Approximately 10–30% of obese individuals are associated with metabolically healthy obese (MHO) phenotype [11–13]. In addition, there is a subgroup of individuals with abnormal metabolic parameters who are not obese, called metabolically unhealthy nonobese (MUNO) or metabolically obese normal weight (MONW) [14, 15]. Combining obesity with different metabolic profiles, obesity phenotypes are better predictors of cardiovascular disease and mortality than obesity per se [16]. Different obesity phenotypes may also help us to understand whether obesity per se or coexisting metabolic abnormalities can increase the risk of hypothyroidism.

Only a few previous studies have investigated whether thyroid function could identify obesity phenotypes in euthyroid subjects [17–20]. However, no cohort studies have investigated the relationship between different obesity phenotypes and the development of hypothyroidism. In addition, since thyroid function and disorders often present differently in different sex and age groups, whether this relationship is modified by sex and age is also worth investigating. In the present study, we aimed to assess the sex-specific association between different obesity phenotypes and the development of hypothyroidism, as well as the modifying role of age.

## Materials and methods

### Study population

**Fig. 1:**
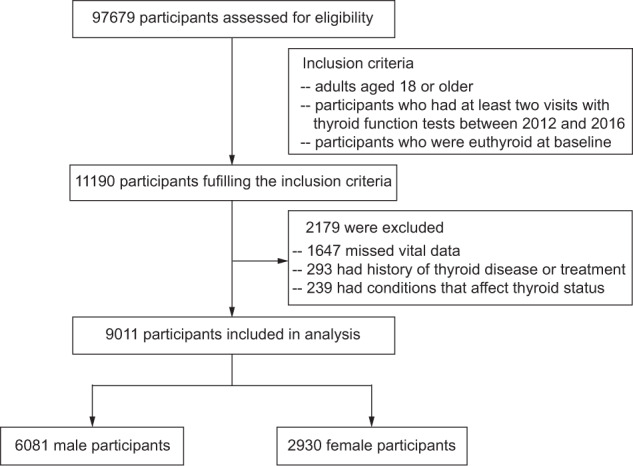
The flow chart of enrollment

This was a retrospective cohort study. The study population was derived from the health management cohort composed of individuals who underwent a comprehensive health examination in Shandong Provincial Hospital. The inclusion criteria were as follows: (1) adults aged 18 or older; (2) participants who had at least two visits with thyroid function tests between 2012 and 2016; and (3) participants with normal thyroid function at baseline. Of 97,679 participants who underwent health examination in Shandong Provincial Hospital between 2012 and 2016, 11,190 individuals fulfilling the inclusion criteria were examined for eligibility. Our exclusion criteria were as follows: (1) missing vital data, such as age and data necessary for categorization of obesity phenotype; (2) self-reported history of thyroid disease, use of medication that influences thyroid function, radioactive iodine therapy, or thyroidectomy; and (3) conditions that affect thyroid status during the study period, such as pregnancy, severe hepatic or renal dysfunctions, or malignant tumor. Ultimately, 6081 males and 2930 females were included in our study (Fig. 1). This study was approved by the ethics committee of Shandong Provincial Hospital Affiliated to Shandong University (LCYJ: NO. 2019-002).

### Clinical and laboratory measurements

The data were collected from the health management database in Shandong Provincial Hospital. Information on medical history and lifestyle was gathered by a standardized questionnaire. After an overnight fast of at least 10 h, each subject underwent a standardized medical examination, including anthropometric and laboratory tests. Height and weight were measured after participants removed their shoes and heavy clothing. Body mass index (BMI) was calculated as weight (kg)/height squared (m^2^). Blood pressure was measured twice after a 5–15 min rest period and the mean values were reported. Thyroid-stimulating hormone (TSH), free thyroxine (FT4), and free triiodothyronine (FT3) were measured by chemiluminescent methods (Cobas E601; Roche, Basel, Switzerland). Thyroperoxidase antibodies (TPO-Ab) were measured using ADVIA Centaur XP (Siemens, Germany). Laboratory tests also included serum glucose, lipid profile, hepatic function, and renal function measured by the ARCHITECT ci16200 Integrated System (Abbott, Illinois, USA).

### Definitions

Obesity was defined as BMI ≥ 25 kg/m^2^, which has been recommended as a more reasonable threshold to define obesity for Asians [21–23]. According to the diagnostic criteria for metabolic syndrome of the Chinese Diabetes Society [24], metabolically unhealthy was defined as having at least two of the following four metabolic syndrome components: (1) fasting plasma glucose (FPG) ≥ 6.1 mmol/L; (2) systolic blood pressure (SBP) ≥ 130 mmHg or diastolic blood pressure (DBP) ≥ 85 mmHg; (3) triglycerides (TGs) ≥ 1.7 mmol/L; and (4) high-density lipoprotein cholesterol (HDL-C) < 1.0 mmol/L. Waist circumference was excluded from the definitions of metabolically unhealthy status due to its high multicollinearity with BMI [20]. According to their obesity and metabolic statuses, individuals were classified into four different obesity phenotypes: (1) metabolically healthy nonobese (MHNO): BMI < 25 kg/m^2^ and fewer than two metabolic syndrome components; (2) MHO: BMI ≥ 25 kg/m^2^ and fewer than two metabolic syndrome components; (3) MUNO: BMI < 25 kg/m^2^ and at least two metabolic syndrome components; and (4) metabolically unhealthy obese (MUO): BMI ≥ 25 kg/m^2^ and at least two metabolic syndrome components.

The reference ranges were 0.27–4.2 mIU/L for TSH, 12–22 pmol/L for FT4, 3.1–6.8 pmol/L for FT3, and 0–60 IU/L for TPO-Ab. Euthyroidism was defined as serum TSH, FT4, and FT3 levels within the reference ranges. Hypothyroidism was defined as TSH > 4.2 mIU/L and FT4 < 12 pmol/L (overt hypothyroidism) or TSH > 4.2 mIU/L and FT4 levels within the reference range (subclinical hypothyroidism).

### Statistical analysis

Continuous variables with a normal distribution are presented as the mean (standard deviation), and continuous variables with a skewed distribution were presented as the median [25th percentile, 75th percentile]. Categorical variables are presented as numbers (percentages). Differences in continuous variables were compared by using one-way analysis of variance or Kruskal–Wallis one-way analysis of variance. Bonferroni correction was applied to all multiple comparisons. For categorical variables, the chi-square test or Fisher’s exact test was used for group comparisons. The incidence density of hypothyroidism was calculated using the number of incident cases of hypothyroidism and person-years of follow-up in each group. A mid-P exact test was applied to calculate 95% confidence intervals (CIs) and compare incidence densities between groups.

The generalized estimation equation (GEE) method was used to investigate the associations between different metabolic obesity phenotypes and the development of hypothyroidism, which is an extension of the generalized linear model to allow for analysis of repeated measurements or other correlated observations [25]. First introduced by Liang and Zeger, GEE is a widely used and flexible estimation method for longitudinal data, allowing the use of all available information [26]. Instead of using only baseline and outcome information, we make full use of obesity phenotype at each time point by using GEE method. Sensitivity analyses were conducted by excluding participants with positive TPO-Ab at baseline. The odds ratios (ORs) and 95% CIs for different metabolic obesity phenotypes in association with hypothyroidism were reported. A two-tailed *p* value < 0.05 was considered statistically significant. All statistical analyses were performed in RStudio (version 1.0.143) with R (version 4.0.0).

## Results

### Baseline characteristics of males and females based on obesity phenotype

Of the 97,679 participants who underwent health examination in Shandong Provincial Hospital between 2012 and 2016, 11,190 adults who were euthyroid at baseline and had thyroid function tests at each visit were examined for eligibility. A total of 9011 participants were included in the final analysis, of whom 6081 were men (Fig. 1). The median follow-up time was 1.92 (1.00–2.17) years. Based on obesity phenotype at baseline, in both males and females, the MHNO group was the largest group of the four obesity phenotype groups (Supplementary Fig. 1a). The proportions of MHO, MUNO, and MUO were higher in males than in females (all *p* < 0.001) (Supplementary Fig. 1a). The proportions of the MUNO and MUO phenotypes were higher in older males than in younger males (cutoff of 55 years, all *p* < 0.001) (Supplementary Fig. 1b). In older females, the proportions of the MHO, MUNO, and MUO phenotypes were higher than those in younger females (cutoff of 55 years all *p* < 0.001) (Supplementary Fig. 1c). The numbers of participants with the different obesity phenotypes during follow-up are shown in Supplementary Fig. 2.

The baseline characteristics of males (*N* = 6081) based on obesity phenotypes are summarized in Table 1. The average age of males was 50.53 (14.11) years. Individuals with the MUNO and MUO phenotypes had significantly higher TGs, SBP, DBP, and FPG, and lower HDL-C than individuals with the MHNO and MHO phenotypes (all *p* < 0.05). Individuals with the MHO and MUO phenotypes had higher BMI and alanine aminotransferase (ALT) levels than individuals with the MHNO and MUNO phenotypes (all *p* < 0.001). Regarding thyroid function, baseline serum TSH levels, FT4 levels, and TPO-Ab positivity were comparable between the four groups, while individuals with the MHO and MUO phenotypes had significantly higher FT3 than the MHNO participants (all *p* < 0.05). In addition, baseline TC, LDL-C, aspartate aminotransferase (AST), creatinine (Cr), estimated glomerular filtration rate (eGFR), and smoking were also significantly different between groups (all *p* < 0.001).

**Table 1:** Baseline characteristics of male participants based on different metabolic obesity phenotypes

	Overall (*N* = 6081)	MHNO (*N* = 2001)	MHO (*N* = 1989)	MUNO (*N* = 551)	MUO (*N* = 1540)	*p* value
Age (years)	50.53 (14.11)	49.06 (15.15)	48.95 (12.42)	57.15 (15.25)^ab^	52.12 (13.50)^abc^	<0.001
BMI (kg/m^2^)	25.69 (2.97)	22.87 (1.65)	27.31 (1.90)^a^	23.52 (1.17)^ab^	28.03 (2.29)^abc^	<0.001
TC (mmol/L)	5.23 (0.93)	5.11 (0.90)	5.25 (0.88)^a^	5.25 (0.94)^a^	5.35 (1.00)^ab^	<0.001
TG (mmol/L)	1.40 [0.99, 2.00]	1.07 [0.82, 1.41]	1.29 [1.00, 1.63]^a^	1.95 [1.39, 2.52]^ab^	2.09 [1.65, 2.80]^abc^	<0.001
HDL-C (mmol/L)	1.26 (0.29)	1.38 (0.30)	1.27 (0.23)^a^	1.17 (0.31)^ab^	1.11 (0.25)^abc^	<0.001
LDL-C (mmol/L)	3.03 (0.76)	2.93 (0.75)	3.13 (0.73)^a^	2.97 (0.77)^b^	3.05 (0.82)^ab^	<0.001
SBP (mmHg)	127.57 (17.19)	119.96 (15.04)	124.78 (14.63)^a^	135.97 (17.15)^ab^	138.05 (16.45)^abc^	<0.001
DBP (mmHg)	74.26 (11.26)	69.40 (9.81)	73.79 (10.20)^a^	76.16 (11.13)^ab^	80.49 (11.24)^abc^	<0.001
FPG (mmol/L)	5.35 [4.98, 5.89]	5.16 [4.85, 5.52]	5.24 [4.93, 5.60]^a^	5.93 [5.30, 6.86]^ab^	5.96 [5.27, 6.90]^ab^	<0.001
TSH (IU/mL)	1.76 [1.29, 2.37]	1.74 [1.27, 2.34]	1.79 [1.31, 2.37]	1.82 [1.27, 2.40]	1.75 [1.30, 2.41]	0.329
FT4 (pmol/L)	16.63 (1.97)	16.68 (1.96)	16.60 (2.00)	16.54 (1.91)	16.63 (1.96)	0.407
FT3 (pmol/L)	5.19 (0.52)	5.16 (0.51)	5.23 (0.51)^a^	5.11 (0.55)^b^	5.21 (0.52)^ac^	<0.001
TPO-Ab positive (%)	480 (7.9)	147 (7.3)	163 (8.2)	48 (8.7)	122 (7.9)	0.664
ALT (IU/L)	23 [17, 31]	20 [15, 26]	24 [18, 32]^a^	22 [17, 28]^ab^	26 [19, 36]^abc^	<0.001
AST (IU/L)	22 [19, 26]	21 [19, 25]	22 [19, 26]^a^	22 [19, 26]^a^	23 [19, 28]^abc^	<0.001
Cr (μmol/L)	87.46 (13.10)	87.37 (13.03)	88.89 (12.62)^a^	85.00 (13.36)^ab^	86.60 (13.48)^b^	<0.001
eGFR (mL/min/1.73m^2^)	87.80 [77.56, 102.47]	88.22 [78.38, 103.68]	86.57 [76.53, 99.51]^a^	89.59 [78.90, 103.75]^b^	88.54 [77.37, 103.34]^b^	<0.001
Smoking (%)	2264 (37.2)	669 (33.4)	763 (38.4)^a^	208 (37.7)	624 (40.5)^a^	<0.001

*MHNO* metabolically healthy nonobese, *MHO* metabolically healthy obese, *MUNO* metabolically unhealthy nonobese, *MUO* metabolically unhealthy obese, *BMI* body mass index, *TC* total cholesterol, *TG* triglyceride, *HDL-C* high-density lipoprotein cholesterol, *LDL-C* low-density lipoprotein cholesterol, *SBP* systolic blood pressure, *DBP* diastolic blood pressure, *FPG* fasting plasma glucose, *TSH* thyroid-stimulating hormone, *FT4* free thyroxine, *FT3* free triiodothyronine, *TPO-Ab* thyroid peroxidase antibody, *ALT* alanine aminotransferase, *AST* aspartate aminotransferase, *Cr* creatinine, *eGFR* estimated glomerular filtration rate, *SD* standard deviation

Values with normal distribution are presented as mean (SD), and values without normal distribution are presented as median [25th percentile, 75th percentile]; categorical variables are presented as *N* (%). Statistically significant differences are shown with superscript letters: ^a^Significant difference compared with MHNO phenotype. ^b^Significant difference compared with MHO phenotype. ^c^Significant difference compared with MUNO phenotype

Baseline characteristics of females (*N* = 2930) based on obesity phenotypes are summarized in Supplementary Table 1. The average age of females was 46.56 (14.24) years. TC, TGs, LDL-C, SBP, DBP, FPG, and AST in the MUNO and MUO groups were significantly higher than those in the MHNO and MHO groups, while BMI was higher in the MHO and MUO groups (all *p* < 0.05). Baseline serum TSH levels, FT4 levels, and FT3 levels were comparable between the four groups. In addition, there were significant differences in baseline TPO-Ab positivity, HDL-C, ALT, Cr, eGFR, and smoking between groups (all *p* < 0.05).

### The non-MHNO group had a significantly higher incidence density of hypothyroidism than the MHNO group in males

**Fig. 2:**
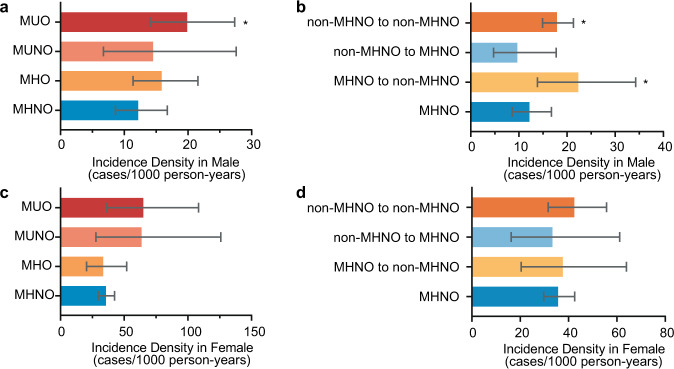
The incidence density and 95% CI of hypothyroidism based on different obesity phenotypes in male and female participants. **a** The incidence density of hypothyroidism in male participants with consistent obesity phenotypes during follow-up. **b** The incidence density of hypothyroidism based on the change of obesity phenotypes during follow-up in all male participants. **c** The incidence density of hypothyroidism in female participants with consistent obesity phenotypes during follow-up. **d** The incidence density of hypothyroidism based on the change of obesity phenotypes during follow-up in all female participants. *p < 0.05 compared with MHNO group. MHNO metabolically healthy nonobese, MHO metabolically healthy obese, MUNO metabolically unhealthy nonobese, MUO metabolically unhealthy obese, non-MHNO obesity phenotypes except MHNO (including MHO, MUNO, and MUO)

As the follow-up time was different between groups, we utilized incidence density to assess the number of incident hypothyroidism per unit time in different groups. The incidence densities of hypothyroidism in males with consistent obesity phenotypes during follow-up were 12.19 (8.62–16.76), 15.87 (11.39–21.56), 14.52 (6.74–27.57), and 19.88 (14.06–27.34) per 1000 person-years in the MHNO, MHO, MUNO, and MUO groups, respectively (Fig. 2a). Considering the participants with changes in obesity phenotypes during follow-up, we further analyzed the incidence density of hypothyroidism in different groups based on the change in obesity phenotypes. The incidence densities of hypothyroidism were 22.35 (13.86–34.26), 9.67 (4.71–17.74), and 17.91 (14.93–21.32) per 1000 person-years in the MHNO to non-MHNO (MHO, MUNO, or MUO), non-MHNO to MHNO, and non-MHNO to non-MHNO groups, respectively (Fig. 2b). Compared with the MHNO group, the MUO, MHNO to non-MHNO, and non-MHNO to non-MHNO groups had significantly higher incidence densities (all *p* < 0.05).

The incidence density of hypothyroidism in females was significantly higher than that in males (*p* < 0.001). Among females with consistent obesity phenotypes, the incidence densities of hypothyroidism were 35.66 (29.70–42.49), 33.52 (20.49–51.95), 63.64 (27.83–125.90), and 65.00 (36.15–108.40) per 1000 person-years in the MHNO, MHO, MUNO, and MUO groups, respectively (Fig. 2c). According to the change in obesity phenotypes during follow-up, the incidence densities of hypothyroidism were 37.62 (20.38–63.95), 33.33 (16.26–61.17), and 42.37 (31.59–55.70) per 1000 person-years in the MHNO to non-MHNO, non-MHNO to MHNO, and non-MHNO to non-MHNO groups, respectively (Fig. 2d). In contrast to males, although a trend exists, there was no significant difference between the non-MHNO groups and the MHNO group in females. In summary, the non-MHNO group had a significantly higher incidence density of hypothyroidism than the MHNO group in males.

### MHO, MUNO, and MUO phenotypes were independent risk factors for the development of hypothyroidism compared with the MHNO phenotype in males

**Fig. 3:**
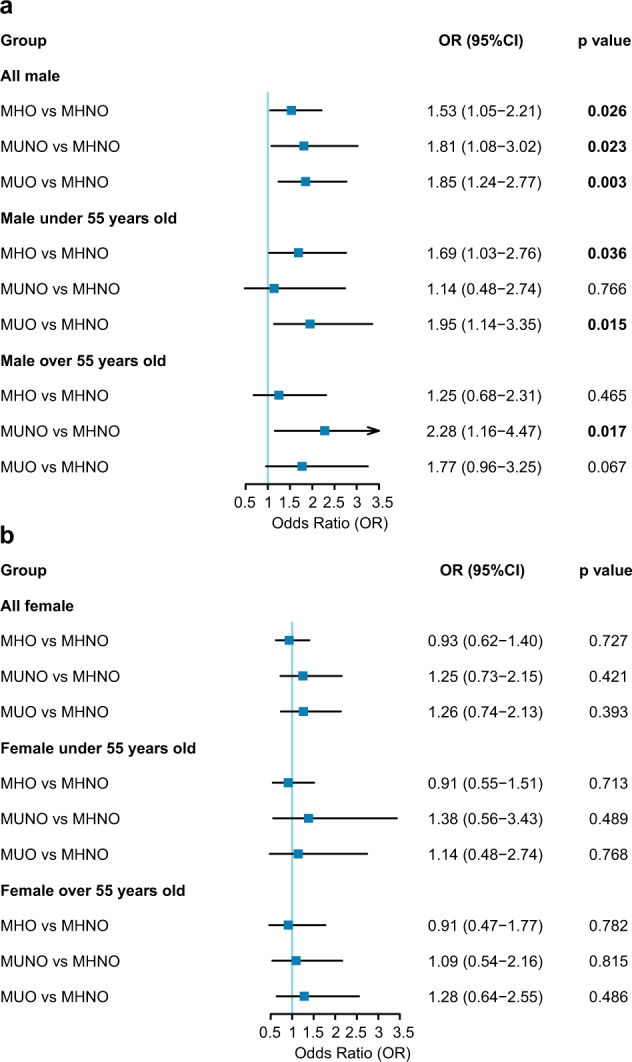
Forest plot for the odds ratio of developing hypothyroidism based on different obesity phenotypes. **a** In male participants. **b** In female participants. Odds ratios (odds of developing hypothyroidism for participants with unhealthy obesity phenotypes compared with participants with MHNO phenotype) are after adjustment for baseline age, follow-up time, TPO-Ab, ALT, Cr, and smoking. MHNO metabolically healthy nonobese, MHO metabolically healthy obese, MUNO metabolically unhealthy nonobese, MUO metabolically unhealthy obese

Considering the change in the obesity phenotype during follow-up and the potential confounding factors associated with thyroid function such as age and TPO-Ab, we utilized GEE analysis to further evaluate the relationship between obesity phenotypes and hypothyroidism in this cohort study. The results of GEE analysis for developing hypothyroidism based on different obesity phenotypes during follow-up according to sex are presented in Table 2 and Fig. 3. These results showed that in males, the MHO, MUNO, and MUO phenotypes were all risk factors for developing hypothyroidism compared with the MHNO phenotype. In the unadjusted model, participants with the MHO phenotype had a 1.52-fold increased odds of developing hypothyroidism (*p* = 0.028), while participants with the MUNO and MUO phenotypes had 2.03-fold (*p* = 0.007) and 1.91-fold (*p* = 0.001) increased odds of developing hypothyroidism, respectively (Table 2). Further adjusting for the potential confounders of age, follow-up time, TPO-Ab, ALT, Cr, and smoking did not change this association (Table 2, Fig. 3a).

**Table 2:** Results of GEE analysis for developing hypothyroidism based on different obesity phenotype during follow-up in male and female participants

	Unadjusted model		Model 1		Model 2		Model 3	
	OR (95% CI)	*p* value	OR (95% CI)	*p* value	OR (95% CI)	*p* value	OR (95% CI)	*p* value
Male								
MHNO	1 (Reference)		1 (Reference)		1 (Reference)		1 (Reference)	
MHO	1.52 (1.05–2.21)	0.028	1.54 (1.06–2.23)	0.022	1.54 (1.06–2.23)	0.023	1.53 (1.05–2.21)	0.026
MUNO	2.03 (1.21–3.41)	0.007	1.82 (1.09–3.02)	0.021	1.82 (1.09–3.02)	0.022	1.81 (1.08–3.02)	0.023
MUO	1.91 (1.28–2.84)	0.001	1.87 (1.25–2.78)	0.002	1.87 (1.26–2.79)	0.002	1.85 (1.24–2.77)	0.003
Female								
MHNO	1 (Reference)		1 (Reference)		1 (Reference)		1 (Reference)	
MHO	1.03 (0.69–1.52)	0.902	0.97 (0.64–1.47)	0.897	0.97 (0.64–1.47)	0.893	0.93 (0.62–1.40)	0.727
MUNO	1.59 (0.95–2.66)	0.076	1.32 (0.78–2.26)	0.302	1.34 (0.78–2.29)	0.284	1.25 (0.73–2.15)	0.421
MUO	1.50 (0.93–2.41)	0.093	1.36 (0.80–2.30)	0.257	1.38 (0.82–2.32)	0.232	1.26 (0.74–2.13)	0.393

Model 1: adjustment for age and follow-up time. Model 2: adjustment for age, follow-up time and TPO-Ab. Model 3: adjustment for age, follow-up time, TPO-Ab, ALT, Cr, and smoking

*MHNO* metabolically healthy nonobese, *MHO* metabolically healthy obese, *MUNO* metabolically unhealthy nonobese, *MUO* metabolically unhealthy obese, *TPO-Ab* thyroid peroxidase antibody, *GEE* generalized estimating equations, *OR* odds ratio, *CI* confidence interval

Contrasting the findings among male participants, non-MHNO phenotypes were not independent risk factors for the development of hypothyroidism in female participants. Although female participants with the MUNO and MUO phenotypes showed a trend toward being more likely to develop hypothyroidism in the unadjusted model, the association no longer remained after adjustment for confounders (Table 2, Fig. 3b). Sensitivity analyses showed that findings remained unchanged when participants with positive TPO-Ab at baseline were excluded (Supplementary Fig. 3). In summary, the MHO, MUNO, and MUO phenotypes were independent risk factors for the development of hypothyroidism compared with the MHNO phenotype in males, while such an association was not found in females.

### The relationship between obesity phenotypes and hypothyroidism differs according to age groups in males

As previous studies have found that thyroid function changes with age and the association between thyroid dysfunction and metabolic abnormalities differs according to age [27, 28], we examined the relationship between obesity phenotypes and hypothyroidism in younger and older participants separately (cutoff of 55 years). Among younger males, the MHO and MUO phenotypes were associated with a higher risk of developing hypothyroidism than the MHNO phenotype (MHO: OR 1.79, 95% CI 1.12–2.88; MUO: OR 2.11, 95% CI 1.24–3.57). The association remained in different models adjusting for potential confounding factors (Table 3, Fig. 3a).

**Table 3:** GEE analysis for developing hypothyroidism based on different obesity phenotype during follow-up in male participants according to age (cutoff of 55 years)

	Unadjusted model		Model 1		Model 2		Model 3	
	OR (95% CI)	*p* value	OR (95% CI)	*p* value	OR (95% CI)	*p* value	OR (95% CI)	*p* value
Male under 55 years old								
MHNO	1 (Reference)		1 (Reference)		1 (Reference)		1 (Reference)	
MHO	1.79 (1.12–2.88)	0.016	1.76 (1.08–2.86)	0.023	1.74 (1.07–2.83)	0.025	1.69 (1.03–2.76)	0.036
MUNO	1.31 (0.54–3.21)	0.549	1.19 (0.49–2.85)	0.701	1.18 (0.49–2.83)	0.714	1.14 (0.48–2.74)	0.766
MUO	2.11 (1.24–3.57)	0.006	2.06 (1.21–3.51)	0.008	2.05 (1.20–3.50)	0.008	1.95 (1.14–3.35)	0.015
Male over 55 years old								
MHNO	1 (Reference)		1 (Reference)		1 (Reference)		1 (Reference)	
MHO	1.14 (0.61–2.13)	0.674	1.24 (0.67–2.28)	0.496	1.24 (0.67–2.28)	0.495	1.25 (0.68–2.31)	0.465
MUNO	2.30 (1.18–4.47)	0.014	2.19 (1.12–4.27)	0.022	2.19 (1.12–4.27)	0.022	2.28 (1.16–4.47)	0.017
MUO	1.66 (0.91–3.04)	0.102	1.70 (0.93–3.13)	0.086	1.71 (0.93–3.14)	0.086	1.77 (0.96–3.25)	0.067

Model 1: adjustment for age and follow-up time. Model 2: adjustment for age, follow-up time, and TPO-Ab. Model 3: adjustment for age, follow-up time, TPO-Ab, ALT, Cr, and smoking

*MHNO* metabolically healthy nonobese, *MHO* metabolically healthy obese, *MUNO* metabolically unhealthy nonobese, *MUO* metabolically unhealthy obese, *TPO-Ab* thyroid peroxidase antibody, *GEE* generalized estimating equations, *OR* odds ratio, *CI* confidence interval

In contrast to younger males, only the MUNO phenotype was accompanied by a twofold increased risk of developing hypothyroidism among older males in the unadjusted model (OR 2.30, 95% CI 1.18–4.47). Further adjusting for potential confounders did not change this association (Table 3, Fig. 3a). In addition, participants with the MUO phenotype also showed a trend toward an increased risk of developing hypothyroidism (OR 1.77, 95% CI 0.96–3.25). We also performed subgroup analyses in females according to age. Nevertheless, non-MHNO phenotypes were not independent risk factors for developing hypothyroidism in either younger or older females (Supplementary Table 2, Fig. 3b). Sensitivity analyses showed that findings remained unchanged when participants with positive TPO-Ab at baseline were excluded (Supplementary Fig. 3). Thus, age modifies the relationship between obesity phenotypes and hypothyroidism in males.

## Discussion

The present cohort study demonstrated that the non-MHNO group had a significantly higher incidence density of hypothyroidism than the MHNO group in males, and the MHO, MUNO, and MUO phenotypes were independent risk factors for the development of hypothyroidism compared with the MHNO phenotype in males but not in females. In addition, age modifies the relationship between obesity phenotypes and hypothyroidism in males. Compared with the MHNO phenotype, the MHO and MUO phenotypes were independent risk factors for the development of hypothyroidism in younger males, while the MUNO phenotype was an independent risk factor in older males.

To the best of our knowledge, this is the first cohort study investigating the association between different obesity phenotypes and the incidence of hypothyroidism focusing on sex and age differences. A few previous studies have investigated the relationship between thyroid function within the reference range and different obesity phenotypes [18–20]. As there are no universally accepted criteria of obesity phenotypes, we adopted the definition previously used in the Chinese population [20]. However, previous studies in China and Korea were cross-sectional studies [19, 20]. In the only longitudinal study conducted in Tehran, the authors aimed to explore the relationship between thyroid function within the reference range and the development of different obesity phenotypes, which was different from our objective [18]. In addition, the sex and age differences in the association between obesity phenotypes and hypothyroidism are also worth investigating. Our study found that the MHO, MUNO, and MUO phenotypes were independent risk factors for developing hypothyroidism compared with the MHNO phenotype in males, while such an association was not found in females. In addition, we found that age modifies the relationship between obesity phenotypes and hypothyroidism in males.

The most common cause of hypothyroidism in iodine-sufficient areas is chronic autoimmune thyroiditis [1]. However, a clear clinical association between obesity and thyroid autoimmunity has not been established to date [7]. A previous study on subjects without thyroid autoimmunity at baseline found no significant association between the baseline abdominal obesity phenotype and the development of TPO-Ab positivity [29]. Our study also found that the relationship between obesity phenotypes and hypothyroidism was independent of TPO-Ab positivity. Although the mechanisms underlying the association between the obesity phenotype and hypothyroidism have not yet been elucidated, some explanations have been proposed. In obese subjects, a chronic low-grade inflammatory state is present. The increase in inflammatory cytokines such as interleukin-1 (IL-1), IL-6, and tumor necrosis factor-alpha can inhibit the expression of sodium-iodide symporter (NIS) and influence iodide uptake activity, and may contribute to morphological changes in the thyroid [30, 31]. Leptin may also play a role in suppressing TSH-induced thyroid function in individuals with obesity [32]. In addition, chronic inflammation may also affect thyroid function by modulating deiodinase [33, 34]. Another possible explanation is that the thyroid might be a target organ affected by lipotoxicity [35–39]. Our previous study found that palmitic acid could downregulate the expression and activity of three key molecules in thyroid hormone synthesis (NIS, thyroglobulin, and thyroperoxidase) in human primary thyrocytes [36]. Endoplasmic reticulum stress might play a role in high-fat diet-induced hypothyroidism [38].

It is well known that the prevalence of hypothyroidism is significantly higher in females than in males [2]. Our study also found that females had a significantly higher incidence density of hypothyroidism than males. In addition, some previous studies have suggested sex differences in the association of obesity or metabolic abnormalities with thyroid disease [28, 40–42]. The findings from a cohort study indicated that obesity and metabolic health may differently affect the development of thyroid cancer according to sex [42], while a longitudinal study on sex differences in the association of obesity phenotypes with hypothyroidism is still lacking. Our study found that the MHO, MUNO, and MUO phenotypes were independent risk factors for the development of hypothyroidism in males, whereas such an association was not found in females. Although the mechanisms underlying the sex difference we found in the associations of obesity phenotypes with hypothyroidism are not clear, there are several possible explanations. First, men are more likely to accumulate visceral adipose tissue than women, which means that obesity is more hazardous in men than women [43]. Second, estradiol and testosterone have different effects in regulating thyroid function [44, 45]. Different obesity phenotypes may cause sex-specific changes in sex hormones, which may lead to sex differences in the risk of developing hypothyroidism. Finally, the average age in females with the MUNO and MUO phenotypes was more than 60 years, which means that these two groups were mostly composed of postmenopausal females. The influence of estrogen might mask the relationship between obesity phenotypes and thyroid function. In addition, 2124 (72.5%) females in our study population had the MHNO phenotype, and the sample sizes in the other three groups were relatively small. Our findings suggested that maintaining a normal healthy weight was associated with a lower risk of hypothyroidism in men. Further research is needed to validate our findings and uncover the underlying mechanisms.

Previous studies have reported increased serum TSH levels in older patients and the association between thyroid dysfunction and lipid profiles differs according to age [27, 28]. Thus, we further analyzed the association of obesity phenotypes with hypothyroidism in different age groups. Our results suggested that in younger males, obesity per se was an independent risk factor for hypothyroidism, while in older males only metabolic abnormalities were associated with an increased risk of developing hypothyroidism. The changes in body composition and muscle loss associated with aging could be related to the observed age-specific association. Further studies with body composition data may help to achieve a better understanding of the potential mechanism.

Several limitations should be noted when interpreting the findings from this study. First, this cohort study was an observational study, which is incapable of inferring causal relationships. The relationship between obesity and thyroid function is complex and bidirectional [31]. The thyroid might be a target organ of lipotoxicity [35–39], while thyroid hormones also play important roles in regulating glucose and lipid metabolism [46]. Although we performed a cohort study among baseline euthyroid participants and adjusted for confounding factors, we still cannot exclude potential reverse causation and the influence of unmeasured confounders in the observed relationship. Second, the diagnosis of obesity in our study was based on BMI. Further study with body composition data and waist circumference may give us a better understanding of the association between obesity phenotypes and hypothyroidism. In addition, the diagnosis of hypothyroidism was based on the measured thyroid function at each visit. Last, this cohort was derived from subjects who participated in health examinations regularly in China. The generalizability of our findings to other populations with different characteristics requires further examination.

Despite some limitations, our study had several strengths. First, this was the first cohort study investigating the sex-specific association between different obesity phenotypes and the development of hypothyroidism. Second, our study innovatively found that not only the MUO phenotype but also the MHO and MUNO phenotypes were independent risk factors for the development of hypothyroidism in males, which provides information regarding risk factors for hypothyroidism in males. As hypothyroidism occurs more frequently in females, less attention has been given to males in the past. Our findings suggested that males with unhealthy obesity phenotypes had a higher risk of hypothyroidism, which deserves attention in clinical practice. In addition, we found that the relationship between obesity phenotypes and hypothyroidism differs in different age groups among males. Finally, we used the GEE method for the analysis of the repeated measurements, which makes full use of each observation at each time point, thus improving both the accuracy and credibility of the results.

In conclusion, our results suggested that in the health management cohort, both obesity and metabolic abnormities were associated with a higher risk of hypothyroidism in males. In younger males, excessive adiposity per se was an independent risk factor for hypothyroidism, while in older males metabolic abnormalities were associated with an increased risk of developing hypothyroidism. In contrast to the findings in males, no association between obesity phenotypes and hypothyroidism was observed among females in our population. Our study highlights sex and age differences in the association between obesity phenotypes and the development of hypothyroidism in a baseline euthyroid population, and attention should be paid to males with unhealthy obesity phenotypes. Further research is needed to confirm the generalizability of our findings and elucidate the possible mechanisms underlying the sex and age differences in the association.

## Supplementary information

Supplementary Information
