# Supplementary material for: Association between different obesity phenotypes and hypothyroidism: a study based on a longitudinal health management cohort
Source: Endocrine. 2021 Apr 5;72(3):688–98. doi: 10.1007/s12020-021-02677-2 (PMC8159820; doi:10.1007/s12020-021-02677-2)
Supplement: Supplementary file 1 — Supplementary Information [file 12020_2021_2677_MOESM1_ESM.docx]

**
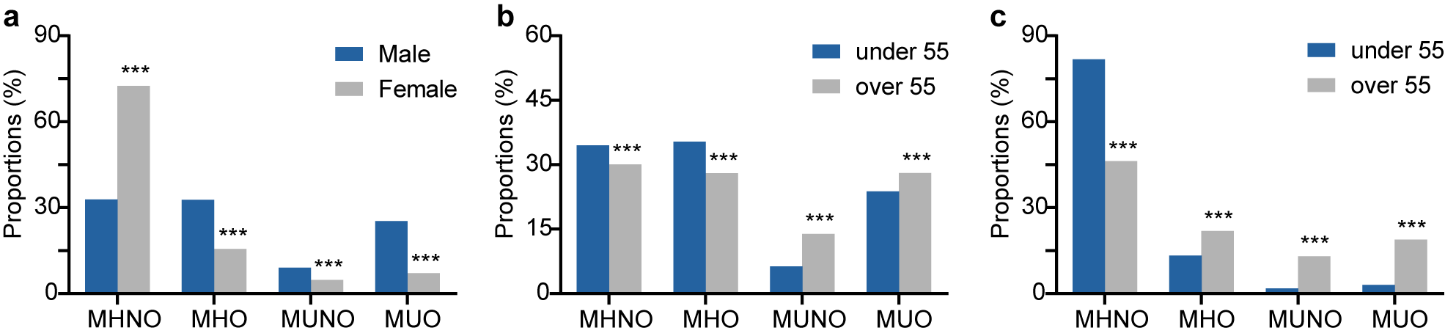
**

**Supplementary Fig. 1** The proportions of participants with different obesity phenotypes at baseline. (a) The proportions of different obesity phenotypes in male and female participants. (b) The proportions of different obesity phenotypes in male participants according to age (cutoff of 55 years). (c) The proportions of different obesity phenotypes in female participants according to age (cutoff of 55 years). *** p<0.001

Abbreviations: MHNO, metabolically healthy nonobese; MHO, metabolically healthy obese; MUNO, metabolically unhealthy nonobese; MUO, metabolically unhealthy obese.


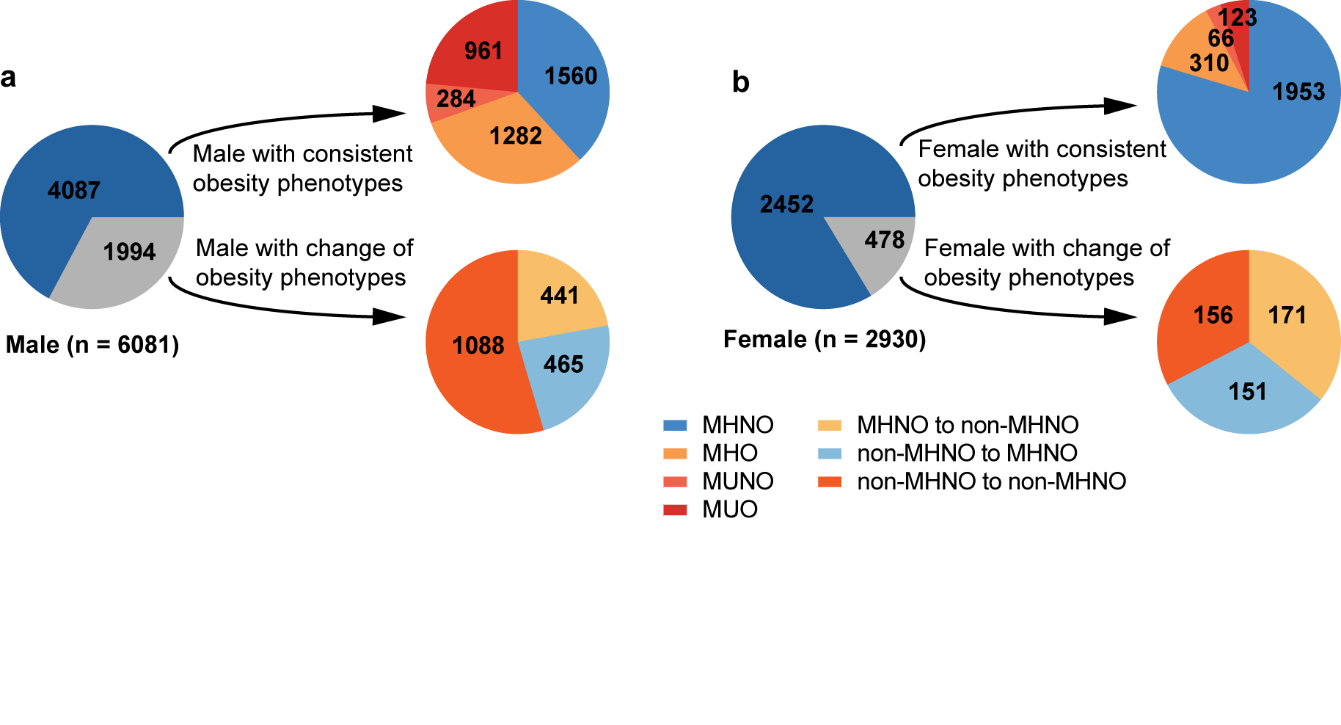


**Supplementary Fig. 2** The number of participants with different obesity phenotypes during follow-up. (a) The number of male participants with different obesity phenotypes during follow-up. (b) The number of female participants with different obesity phenotypes during follow-up.

Abbreviations: MHNO, metabolically healthy nonobese; MHO, metabolically healthy obese; MUNO, metabolically unhealthy nonobese; MUO, metabolically unhealthy obese; non-MHNO, obesity phenotypes except MHNO (including MHO, MUNO and MUO).


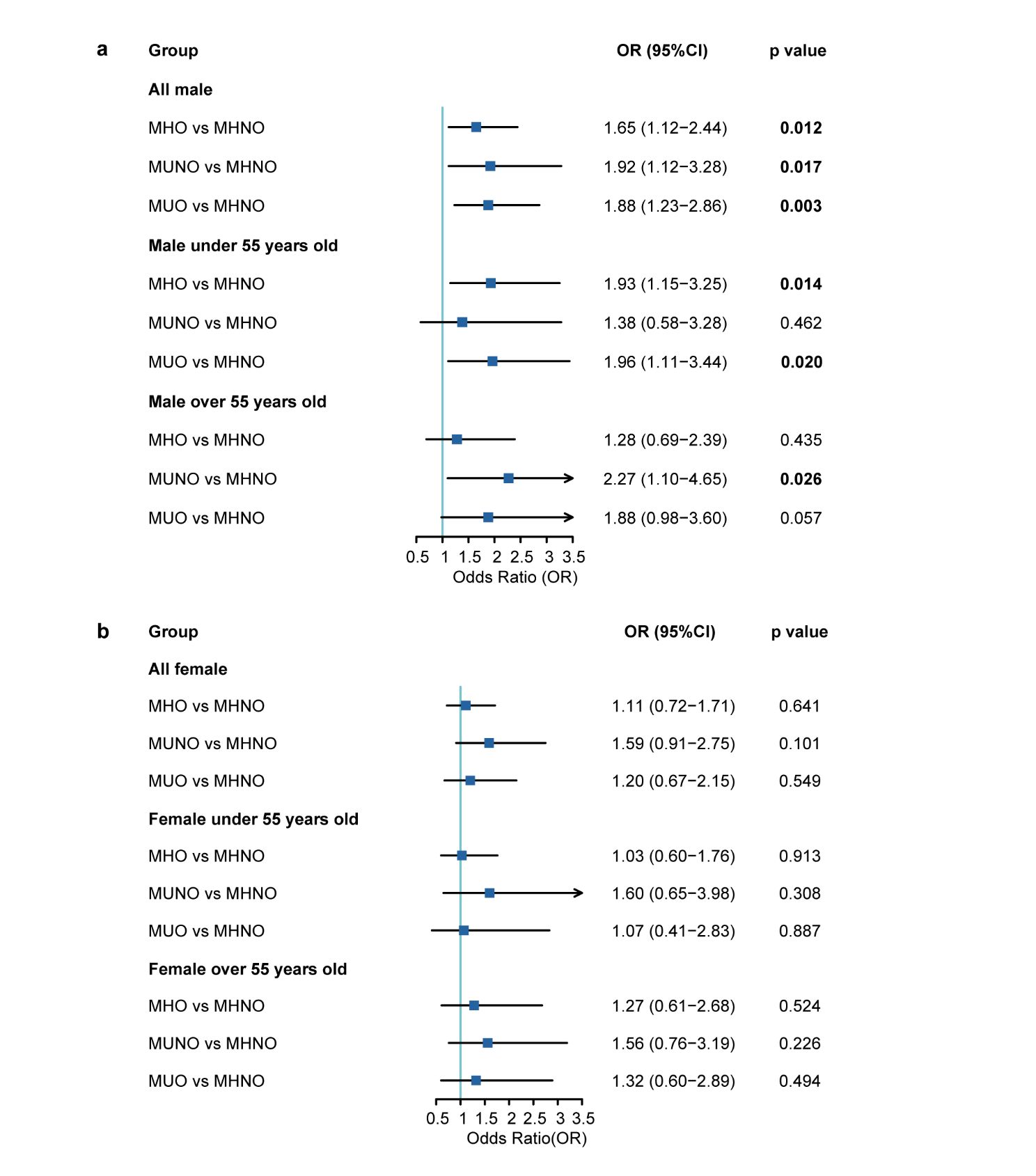


**Supplementary Fig. 3** Forest plot for the odds ratio of developing hypothyroidism based on different obesity phenotypes after excluding participants with positive TPOAb at baseline (a) in male participants. (b) in female participants. Odds ratios (odds of developing hypothyroidism for participants with unhealthy obesity phenotypes compared with participants with MHNO phenotype) are after adjustment for baseline age, follow-up time, TPO-Ab, ALT, Cr and smoking.

Abbreviations: MHNO, metabolically healthy nonobese; MHO, metabolically healthy obese; MUNO, metabolically unhealthy nonobese; MUO, metabolically unhealthy obese.

**Supplementary Table 1** Baseline characteristics of female participants based on different metabolic obesity phenotypes

|  | **Overall**  (N = 2930) | **MHNO**  (N = 2124) | **MHO**  (N = 456) | **MUNO**  (N = 140) | **MUO**  (N = 210) | ***p* Value** |
| --- | --- | --- | --- | --- | --- | --- |
| age (years) | 46.56 (14.24) | 43.14 (12.50) | 50.76 (13.94)^a^ | 62.66 (13.36)^ab^ | 61.36 (12.61)^ab^ | <0.001 |
| BMI (kg/m^2^) | 23.02 (3.09) | 21.64 (1.94) | 27.17 (1.93)^a^ | 23.08 (1.63)^ab^ | 27.95 (2.15)^abc^ | <0.001 |
| TC (mmol/L) | 5.24 (1.02) | 5.11 (0.96) | 5.33 (0.95)^a^ | 6.13 (1.18)^ab^ | 5.84 (1.05)^abc^ | <0.001 |
| TG (mmol/L) | 0.95 [0.70, 1.37] | 0.85 [0.66, 1.14] | 1.13 [0.88, 1.43]^a^ | 1.93 [1.60, 2.35]^ab^ | 2.06 [1.70, 2.54]^ab^ | <0.001 |
| HDL-C (mmol/L) | 1.56 (0.35) | 1.61 (0.35) | 1.46 (0.32)^a^ | 1.45 (0.35)^a^ | 1.30 (0.28)^abc^ | <0.001 |
| LDL-C (mmol/L) | 2.86 (0.81) | 2.73 (0.77) | 3.05 (0.75)^a^ | 3.45 (0.95)^ab^ | 3.37 (0.85)^ab^ | <0.001 |
| SBP (mmHg) | 115.89 (19.31) | 110.02 (14.94) | 122.29 (18.26)^a^ | 142.77 (18.65)^ab^ | 143.49 (17.71)^ab^ | <0.001 |
| DBP (mmHg) | 66.91 (10.68) | 64.69 (9.41) | 69.97 (10.71)^a^ | 74.31 (12.68)^ab^ | 77.78 (10.78)^abc^ | <0.001 |
| FPG (mmol/L) | 5.07 [4.74, 5.48] | 4.97 [4.68, 5.29] | 5.23 [4.93, 5.58]^a^ | 6.26 [5.44, 6.99]^ab^ | 6.27 [5.65, 7.23]^ab^ | <0.001 |
| TSH (IU/ml) | 2.01 [1.49, 2.70] | 1.99 [1.46, 2.69] | 1.96 [1.49, 2.68] | 2.07 [1.52, 2.80] | 2.19 [1.57, 2.90] | 0.110 |
| FT4 (pmol/L) | 15.63 (1.83) | 15.68 (1.83) | 15.51 (1.84) | 15.54 (1.82) | 15.47 (1.78) | 0.128 |
| FT3 (pmol/L) | 4.72 (0.48) | 4.72 (0.48) | 4.76 (0.47) | 4.71 (0.49) | 4.73 (0.44) | 0.354 |
| TPO-Ab positive (%) | 336 (11.5) | 226 (10.6) | 70 (15.4) ^a^ | 16 (11.4) | 24 (11.4) | 0.042 |
| ALT (IU/L) | 15 [12, 20] | 15 [11, 19] | 17 [13, 23]^a^ | 19 [14, 24]^a^ | 20 [15, 28]^ab^ | <0.001 |
| AST (IU/L) | 20 [17, 23] | 19 [16, 23] | 21 [17, 24]^a^ | 22 [19, 26]^ab^ | 22 [18, 26]^ab^ | <0.001 |
| Cr (μmol/L) | 66.41 (11.30) | 65.86 (11.13) | 67.73 (11.70)^a^ | 68.32 (11.34) | 67.78 (11.69) | <0.001 |
| eGFR (mL/min/1.73m^2^) | 98.74 [86.43, 119.10] | 101.08 [88.45, 121.94] | 95.66 [81.89, 115.19]^a^ | 90.50 [81.33, 104.86]^a^ | 92.53 [82.58, 107.06]^a^ | <0.001 |
| smoking (%) | 12 (0.4) | 5 (0.2) | 3 (0.7) | 2 (1.4) | 2 (1.0) | 0.028 |

Abbreviations: MHNO, metabolically healthy nonobese; MHO, metabolically healthy obese; MUNO, metabolically unhealthy nonobese; MUO, metabolically unhealthy obese. BMI, body mass index; TC, total cholesterol; TG, triglyceride; HDL-C, high-density lipoprotein cholesterol; LDL-C, low-density lipoprotein cholesterol; SBP, systolic blood pressure; DBP, diastolic blood pressure; FPG, fasting plasma glucose; TSH, thyroid stimulating hormone; FT4, free thyroxine; FT3, free triiodothyronine; TPO-Ab, thyroid peroxidase antibody; ALT, alanine aminotransferase; AST, aspartate aminotransferase; Cr, creatinine; eGFR, estimated glomerular filtration rate; SD, standard deviation.

Values with normal distribution are presented as mean (SD), and values without normal distribution are presented as median [25^th^ percentile, 75^th^ percentile]; categorical variables are presented as n (%). Statistically significant differences are shown with superscript letters: ^a^ Significant difference compared with MHNO phenotype; ^b^ Significant difference compared with MHO phenotype; ^c^ Significant difference compared with MUNO phenotype.

**Supplementary Table 2** GEE analysis for developing hypothyroidism based on different obesity phenotype during follow-up in female participants according to age (cutoff of 55 years)

|  | **Unadjusted Model** | | **Model 1** | | **Model 2** | | **Model 3** | |
| --- | --- | --- | --- | --- | --- | --- | --- | --- |
|  | OR (95% CI) | *p* Value | OR (95% CI) | *p* Value | OR (95% CI) | *p* Value | OR (95% CI) | *p* Value |
| **Female under 55 years old** | | | | | | | | |
| MHNO | 1 (Reference) |  | 1 (Reference) |  | 1 (Reference) |  | 1 (Reference) |  |
| MHO | 1.02 (0.63-1.67) | 0.924 | 0.96 (0.58-1.60) | 0.879 | 0.97 (0.58-1.61) | 0.893 | 0.91 (0.55-1.51) | 0.713 |
| MUNO | 1.83 (0.75-4.50) | 0.187 | 1.48 (0.61-3.57) | 0.383 | 1.52 (0.63-3.69) | 0.352 | 1.38 (0.56-3.43) | 0.489 |
| MUO | 1.41 (0.61-3.24) | 0.424 | 1.33 (0.56-3.13) | 0.518 | 1.32 (0.56-3.12) | 0.526 | 1.14 (0.48-2.74) | 0.768 |
| **Female over 55 years old** | | | | | | | | |
| MHNO | 1 (Reference) |  | 1 (Reference) |  | 1 (Reference) |  | 1 (Reference) |  |
| MHO | 0.87 (0.44-1.73) | 0.697 | 0.89 (0.45-1.74) | 0.726 | 0.89 (0.45-1.75) | 0.735 | 0.91 (0.47-1.77) | 0.782 |
| MUNO | 1.15 (0.59-2.25) | 0.679 | 1.11 (0.56-2.19) | 0.768 | 1.11 (0.56-2.21) | 0.762 | 1.09 (0.54-2.16) | 0.815 |
| MUO | 1.20 (0.61-2.37) | 0.590 | 1.28 (0.64-2.56) | 0.487 | 1.30 (0.65-2.60) | 0.455 | 1.28 (0.64-2.55) | 0.486 |

Abbreviations: MHNO, metabolically healthy nonobese; MHO, metabolically healthy obese; MUNO, metabolically unhealthy nonobese; MUO, metabolically unhealthy obese; TPO-Ab, thyroid peroxidase antibody; GEE, generalized estimating equations; OR, odds ratio; CI, confidence interval.

Model 1: adjustment for age and follow-up time. Model 2: adjustment for age, follow-up time and TPO-Ab. Model 3: adjustment for age, follow-up time, TPO-Ab, ALT, Cr and smoking.
